# 

**Published:** 1825-03

**Authors:** 


### MONTHLY CATALOGUE OF MEDICAL BOOKS.

* * *It having been hinted to us that gentlemen, sending copies of their Works, prefer having their titles given at length, it is our intention, in future, to comply with this suggestion: but it is to be observed, that no books can be entered on this List except those sent to us for the purpose;—finding, in the lists hitherto transmitted, that the names of works have frequently been given as published, which have not appeared for weeks, or even months, after.*

A Treatise on Moxa, as applicable more particularly to Stiff Joints; illustrated by Cases and Plates: with some general Observations on Spinal Disease. By James Boyle, Esq. Surgeon of the Middlesex Infirmary, &c. &c.—London, 1825.

Elements of Medical Jurisprudence. By Theodric Romeyn Beck, M.D. Professor of the Institutes of Medicine, and Lecturer on Medical Jurisprudence in the College of the Western District of the State of New York, &c. &c. Second Edition, with Notes, and an Appendix of original Cases and the latest Discoveries. By William Dunlop, Member of the Royal College of Surgeons, London; of the Medico-Chirurgical, and of the Wernerian Society of Natural History, Edinburgh; and Lecturer on Medical Jurisprudence, &c. &c.—London, 1825; pp. 640.

An Essay on Venereal Diseases, and the Uses and Abuses of Mercury in their Treatment. Illustrated by Drawings of the different Forms of Venereal Eruptions. Second Edition. By Richard Carmichael, M.R.I.A. Vice-President of the Royal College of Surgeons in Ireland, and one of the Surgeons of the Richmond Surgical Hospital, Dublin, &c. &c. &c.—London, 1825; pp. 376.

Manuel d'Anatomie Générale, Descriptive et Pathologique, par J. F. Meckel, Professeur d'Anatomie à l'Université de Halle. Traduit de l'Allemand, et augmenté des Faits nouveaux dont la Science s'est enrichie jusqu'a ce Jour, par A. J. L. Jourdan, Membre des Académies Royales de Médecine de Paris et des Sciences de Turin, Chevalier de la Légion d'Honneur, &c. et G. Breschet, Professeur agrégé en exercice, Chef des travaux Anatomiques de la Faculté de Médecine de Paris, Chirurgien en chef de l'Hôpital des Enfans Trouvés, Membre de l'Académie Royale de Médecine de Paris. Tome III.—Paris, 1825.

### NOTICE TO CORRESPONDENTS.

*The Communications of* Mr. GRIFFITHS, Mr. ELLIOT TUDOR, *and* Mr. BROWN, *have been received. We regret that* Mr. NORTH's *continuation of his former Paper came to hand too late for insertion in the present Number.*

